# Supplementary material for: Influential node identification method based on multi-order neighbors and exclusive neighborhood
Source: PLoS One. 2025 Aug 13;20(8):e0330199. doi: 10.1371/journal.pone.0330199 (PMC12349128; doi:10.1371/journal.pone.0330199)
Supplement: Attachment — Submitted filename: Response to Reviewers.doc [file pone.0330199.s002.doc]

**Dear editors and reviewers:**

We are very grateful to your consideration and insightful comments for our manuscript **# *PONE-D-25-05440*** entitled **“Influential node identification method based on multi-order neighbors and exclusive neighborhood”**. Thank you very much for the constructive suggestions and for providing us with an opportunity to improve it. In the following we give point-by-point responses to your comments. All responses to reviewers and corrections to the manuscript have been marked in blue.

**Reviewer #1**

In this study, the authors propose a novel method for identifying influential nodes that are effective for diffusion in complex networks. This method introduces the concept of exclusive neighboring nodes in addition to conventional neighboring nodes. Numerical experiments demonstrate that this approach can identify nodes that facilitate diffusion more effectively than existing methods.

The introduction of exclusive neighborhoods presents a fresh and intriguing perspective. The effectiveness of this concept has also been validated through numerical experiments.

However, several aspects of the study are not fully explained, which necessitates a major revision.

Major Comments:

Q1: *Clarification of logarithm usage in Equations (4) and (5)*

*Please provide a detailed explanation of why logarithms are used in these equations. At a minimum, state the advantages of this approach and the reason behind its implementation.*

Reply: Thank you for the constructive suggestions and comments. According to your suggestions, we have explained in Definition 3 of the text the reasons for using logarithms in Equations (4) and (5). The revised manuscript is as follows (**lines 203–218**).

**Definition 3** (initial influence of neighbor nodes): For node *v_i_*, the initial influence provided by its neighbor node *v_j_* is *NI*(*v_j_*). *NI*(*v_j_*) consists of two parts: the influence Г(*v_j_*) provided by the node *v_j_* itself for *v_i_* and the influence I(*v_j_*) provided by the exclusive neighborhood of the node *v_j_*. Since the MNEN method considers the position of a node and the scale of its neighbors as important factors in measuring the influence provided by the node, the *Ks* value and degree value of the node are introduced when calculating Г(*v_j_*) and I(*v_j_*). However, in actual networks, there is a numerical scale difference between the two. The degree value of a node usually has a larger order of magnitude. If the degree value is directly used for calculation, it will first dominate the calculation result of the influence provided by neighboring nodes, reducing the importance of the position reflected by the *Ks* value; secondly, it will make the influence provided by neighboring nodes have an overly large proportion in the calculation of node influence, weakening the importance of the node's own influence. To avoid these problems, the logarithm of the node's degree is taken when calculating Г(*v_j_*) and I(*v_j_*). The calculation formulas of Г(*v_j_*) and I(*v_j_*) are as follows:

$\Gamma\left( vj \right)=\text{Ks}\left( vj \right)^{\text{lg}d\left( vj \right)/{k_{\text{max}}}}$, (4)

$I\left( vj \right)=\sum_{vr\in\text{EN}\left( vi,vj \right)} {\text{Ks}\left( vr \right)}^{\text{lg}d\left( vr \right)/{k_{\text{max}}}}$. (5)

Q2: *Interpretation of the final formula (Equation 9)*

*The study introduces "exclusive neighboring nodes" as a new perspective for identifying influencers. However, the calculation of this index is quite complex, making it unclear what the final metric truly represents. While specific calculation examples are provided (lines 274–302), it would be more informative to illustrate the network structure of high-ranking influencers based on this metric. In other words, the paper should explicitly clarify which nodes are identified as influencers and how their evaluation is influenced by the introduction of exclusive neighborhoods.*

Reply: Thank you for the constructive suggestions and comments. Based on your suggestions, we have clarified the composition of node influence in the text through Formula (9), added a network structure diagram, and provided a detailed description of the node influence calculation process using node *v*_4_ as an example. We also explained the role of exclusive neighborhoods in calculating node influence. Finally, we used the MNEN method to calculate the influence values of core area nodes *v*_1_, *v*_2_, *v*_3_, and *v*_4_ in the network and obtained the influence sequence of all nodes by writing a program in Python. The revised manuscript is as follows (**lines 283–321**).

As can be seen from Formula (9), in the MNEN method, the influence of a node is determined by its own influence and the influence provided by all of its neighbors. When a node is located at the core of the network, has many neighbors, and its neighboring nodes are relatively important, the influence of the node is greater. In Fig 3, *v*_4_ is an example.

**Fig 3. Schematic Diagram of Network Structure.**

First, calculate *SI*(*v*_4_) of the node *v*_4_ itself. Since *Ks*(*v*_4_)=3 and *d*(*v*_4_)=6, then *SI*(*v*_4_)=*Ks*(*v*_4_)+ *d*(*v*_4_)=9.

Second, calculate the influence that the neighbor nodes of node *v*_4_ have on it. According to formula (6), the influence provided by the neighbor nodes consists of their own influence and the influence of their neighbor nodes. The neighbor node set of *v*_4_ is {*v*_1_, *v*_2_, *v*_3_, *v*_5_, *v*_11_, *v*_12_}. Take node *v*_3_ as an example to illustrate the calculation process of the influence provided by neighboring nodes. The neighbor node set of *v*_3_ is {*v*_1_, *v*_2_, *v*_4_, *v*_9_, *v*_10_}. Then, the common neighbor node set *CN*(*v*_4_, *v*_3_)={*v*_1_, *v*_2_} of *v*_3_ and *v*_4_. Nodes *v*_1_ and *v*_2_ are both neighbors of *v*_4_ and *v*_3_, which leads to the situation of repeated calculation of node influence. Therefore, when calculating the influence provided by node *v*_3_, the common neighbor nodes *v*_1_ and *v*_2_ with *v*_4_ are removed, and only the unique neighbor nodes of *v*_3_ are retained, that is, the exclusive neighborhood *EN*(*v*_4_, *v*_3_) of *v*_3_ relative to *v*_4_ is {*v*_4_, *v*_9_, *v*_10_}. This method can avoid the problem of inaccurate results caused by the repeated calculation of the influence of nodes that are connected to many other nodes. In Fig 3, node *v*_4_ is shown in yellow, one-hop neighbor nodes are shown in orange, and the exclusive neighborhood of one-hop neighbor nodes relative to *v*_4_ (excluding *v*_4_) is shown in blue.

The *Ks* value and degree value of node *v*_3_ are *Ks*(*v*_3_)=3 and *d*(*v*_3_)=5. In Fig 3, the maximum value *k_max_* of all node degrees is 6, then the self-influence of node *v*_3_: $\Gamma\left( v3 \right)=\text{Ks}\left( v3 \right)^{\text{lg}d\left( v3 \right)/{k_{\text{max}}}}=3^{\text{lg}\left( 5 \right)/6}=\text{ 1}\text{.}\text{137}$, the calculation formula of influence provided by the exclusive neighborhood of node *v*_3_ is I(*v*_3_)=2.113. The influence value that *v*_3_ can provide is *NI*(*v*_3_)=Γ(*v*_3_)+I(*v*_3_)=1.137+2.113=3.25. Since the correlation between *v*_3_ and *v*_4_ is (2+1)/23=0.1304, the influence value that *v*_3_ can actually provide is 0.13 * 3.25=0.424. By analogy, calculate the actual influence value that all neighbor nodes of node *v*_4_ can provide: *PNI*(*v*_1_)=0.142, *PNI*(*v*_2_)=0.568, *PNI*(*v*_5_)=0.179, *PNI*(*v*_11_)=0.179, and *PNI*(*v*_12_)=0.09, the influence value of node *v*_4_ is 10.582. The influence values of the core area nodes *v*_1_, *v*_2_, and *v*_3_ in the network were calculated using this method, which are 7.549, 10.581, and 9.625 respectively. More detailed influence values of the nodes can be obtained through a Python program.

Python is used to implement the SIR, BC, CC, EC, K-shell, PR, Gravity, PL, ELKSS, GIN, KBKNR, GSM, ECRM, and MNEN algorithms. Then, obtain the sequences of the first 10 nodes in the model network. Set the SIR model contagion probability α=0.01, and the number of iteration runs t = 1000. The sorting results of each algorithm and SIR are displayed in Table 1.

**Table 1. Algorithm sorting results (the last column is sorted by SIR).**

| **BC** | **CC** | **EC** | **K-shell** | **PR** | **Gravity** | **PL** | **ELKSS** | **GIN** | **KBKNR** | **GSM** | **ECRM** | **MNEN** | **SIR** |
| --- | --- | --- | --- | --- | --- | --- | --- | --- | --- | --- | --- | --- | --- |
| 2 | 2 | 4 | 1 | 2 | 2 | 4 | 2 | 2 | 2 | 2 | 2 | 4 | 4 |
| 6 | 4 | 2 | 2 | 4 | 4 | 2 | 4 | 4 | 4 | 4 | 4 | 2 | 2 |
| 3 | 3 | 3 | 3 | 7 | 3 | 3 | 3 | 3 | 3 | 3 | 3 | 3 | 3 |
| 4 | 6 | 1 | 4 | 3 | 1 | 7 | 1 | 1 | 1 | 1 | 1 | 1 | 1 |
| 7 | 1 | 5 | 5 | 16 | 5 | 8 | 5 | 6 | 7 | 5 | 5 | 7 | 7 |
| 8 | 5 | 11 | 6 | 8 | 6 | 11 | 6 | 5 | 6 | 6 | 6 | 5 | 5 |
| 16 | 8 | 12 | 8 | 6 | 8 | 6 | 11 | 8 | 5 | 8 | 8 | 11 | 6 |
| 5 | 7 | 9 | 9 | 9 | 11 | 5 | 8 | 11 | 8 | 11 | 11 | 9 | 11 |
| 9 | 11 | 10 | 10 | 10 | 9 | 9 | 9 | 9 | 9 | 9 | 9 | 10 | 8 |
| 10 | 9 | 6 | 11 | 11 | 10 | 10 | 10 | 10 | 10 | 10 | 10 | 6 | 10 |

Q3: *Clarification of red and blue numbers in the tables*

*I could not find an explanation for the meaning of the red and blue numbers in Tables 1, 3, 4, and 5. Please specify what these color distinctions represent.*

Reply: Thank you. According to your suggestions, we have provided explanations for the meanings of the red and blue numbers in Tables 1, 3, 4, and 5 in the text. The revised manuscript is as follows:

**Table 1. Algorithm sorting results (the last column is sorted by SIR).**

| **BC** | **CC** | **EC** | **K-shell** | **PR** | **Gravity** | **PL** | **ELKSS** | **GIN** | **KBKNR** | **GSM** | **ECRM** | **MNEN** | **SIR** |
| --- | --- | --- | --- | --- | --- | --- | --- | --- | --- | --- | --- | --- | --- |
| 2 | 2 | 4 | 1 | 2 | 2 | 4 | 2 | 2 | 2 | 2 | 2 | 4 | 4 |
| 6 | 4 | 2 | 2 | 4 | 4 | 2 | 4 | 4 | 4 | 4 | 4 | 2 | 2 |
| 3 | 3 | 3 | 3 | 7 | 3 | 3 | 3 | 3 | 3 | 3 | 3 | 3 | 3 |
| 4 | 6 | 1 | 4 | 3 | 1 | 7 | 1 | 1 | 1 | 1 | 1 | 1 | 1 |
| 7 | 1 | 5 | 5 | 16 | 5 | 8 | 5 | 6 | 7 | 5 | 5 | 7 | 7 |
| 8 | 5 | 11 | 6 | 8 | 6 | 11 | 6 | 5 | 6 | 6 | 6 | 5 | 5 |
| 16 | 8 | 12 | 8 | 6 | 8 | 6 | 11 | 8 | 5 | 8 | 8 | 11 | 6 |
| 5 | 7 | 9 | 9 | 9 | 11 | 5 | 8 | 11 | 8 | 11 | 11 | 9 | 11 |
| 9 | 11 | 10 | 10 | 10 | 9 | 9 | 9 | 9 | 9 | 9 | 9 | 10 | 8 |
| 10 | 9 | 6 | 11 | 11 | 10 | 10 | 10 | 10 | 10 | 10 | 10 | 6 | 10 |

According to Table 1 in the example network, the MNEN algorithm identified nodes *v*_4_, *v*_2_, *v*_1_, and *v*_3_ as having relatively high influence. This table presents the top 10 nodes identified by the SIR model and various methods. Nodes in the SIR column are marked in red; if the top 10 node sequence identified by a method is exactly the same as that of SIR, the corresponding nodes are also marked in red; if the top 10 nodes identified by a method exist in SIR's top 10 but the sequence is not the same, the nodes are marked in blue; nodes that do not enter the SIR top 10 list are not specially marked.

Due to the relatively small scale of the example network, all algorithms achieved relatively satisfactory results. Among the top 10 node sequences of each algorithm and SIR, the first 6 nodes of the MNEN method were consistent with the SIR sequence, with only 1 node not appearing in the top 10 nodes of the SIR sequence. In the EC method, the first 4 nodes were consistent with the SIR sequence, but 2 nodes were different from SIR. Although only the first 3 nodes of the PL method were consistent with SIR, only 1 node was different from SIR (**please see lines 321–334 in the revised manuscript**).

Due to space limitations, here we take the Dolphins, USAir, and Powergrid networks as examples. In Tables 3 - 5, the top 10 nodes obtained by all methods and SIR in these three networks are displayed. For consistency, Tables 3 - 5 all adopt the same color-coding rules as Table 1 (**please see lines 430–433 in the revised manuscript**).

*Minor Comments:*

Q4: *Notation consistency in Equation (5)*

*The term EN(v_j) should be written as EN(v_i, v_j) to maintain consistency with the notation used in Equation (3).*

Reply: Thank you very much for your meticulous review and valuable comments on our paper. Due to our oversight, there was an inconsistency in the symbol representation. We have revised Equation (5) in the paper as suggested by you, changing *EN*(*vj*) to *EN*(*vi*, *vj*). The revised manuscript is as follows (**please see line 218 in the revised manuscript**).

$I\left( \text{vj} \right)=\sum_{\text{vr}\in\text{EN}\left( \text{vi},\text{vj} \right)} {\text{Ks}\left( \text{vr} \right)}^{\text{lg}d\left( \text{vr} \right)/{k_{\text{max}}}}$	(5)

Q5: *Notation clarification in line 285*

*The term max ND appears to refer to max k_{max}, but the notation should be aligned for consistency throughout the manuscript.*

Reply: We sincerely appreciate your meticulous review of our paper. Due to our oversight, there was an issue of inconsistent symbols. In accordance with your suggestion, we have revised "max*ND*" to "*k_max_*". The revised manuscript is as follows (**please see line 306 in the revised manuscript**).

$$\Gamma\left( v3 \right)=\text{Ks}\left( v3 \right)^{\text{lg}d\left( v3 \right)/{k_{\text{max}}}}=3^{\text{lg}\left( 5 \right)/6}=\text{ 1}\text{.}\text{137}$$

We hope these revisions address your concerns and improve the overall quality of the manuscript. Thank you again for your valuable feedback.

**Reviewer #2**

This is a really interesting method and a fantastic introduction to the general subject matter. I really enjoyed the introduction and background sections. The method is clearly described; although I think it could be made more concise without losing clarity, I prefer the longer explanation. Small comment there is that the notation "Ks value" for "k-shell" was never introduced.

Q1: *There is that the notation "Ks value" for "k-shell" was never introduced.*

Reply: Thank you for your careful and detailed reading. According to your suggestion, we have added the definition explanation of "*Ks* value" representing "*k-shell*" at the first appearance of "*Ks* value" in the abstract of the article. The revised manuscript is as follows (**lines 9–18**).

In a complex network, the identification of node influence and the localization of key nodes play a crucial role in analyzing network structure and determining the positioning of nodes for information transmission control, resource redistribution, and network regulation. In this study, we propose a method for identifying influential nodes called "Multi-order Neighbors and Exclusive Neighborhood" (MNEN) after analyzing and investigating existing methods in the field. The MNEN method calculates a node's influence based on two factors: the node itself, its neighboring nodes, and its exclusive neighborhood. The influence of the node itself is determined by its degree value and K-shell (*Ks*) value, while the influence contribution of the neighbor node is calculated based on its degree value, *Ks* value, and the contribution from its exclusive neighbor node.

Q2: *My main concern with this work is the level of claims for every result section that this method is "superior", "more consistent", etc. I think a little bit more humility would still leave the method looking great (it seems like a great method!), while acknowledging that other methods out-perform it at times. It was a bit tedious to read an enthusiastic claim at the end of every results paragraph. Highlighting some areas where other models out-perform MNEN would seem appropriate, and generally listing comparison metrics for the other models would seem like better use of space than simply listing all the top nodes for each model.*

Reply: Thank you very much for your insightful and constructive comments. Thank you very much for your insightful and constructive comments. We fully agree with your suggestions and have made the following revisions in accordance with your advice:

(1) In each result section, we now present the comparative performance of the MNEN method in a more objective and comprehensive manner, based strictly on quantitative results. We have removed overly enthusiastic or absolute claims, and instead provided a more nuanced interpretation that acknowledges the strengths and limitations of each method. The revised manuscript is as follows:

Due to the relatively small scale of the example network, all algorithms achieved relatively satisfactory results. Among the top 10 node sequences of each algorithm and SIR, the first 6 nodes of the MNEN method were consistent with the SIR sequence, with only 1 node not appearing in the top 10 nodes of the SIR sequence. In the EC method, the first 4 nodes were consistent with the SIR sequence, but 2 nodes were different from SIR. Although only the first 3 nodes of the PL method were consistent with SIR, only 1 node was different from SIR (**please see lines 329–334 in the revised manuscript**).

According to Table 3, in the Dolphins network, the recognition effects of various algorithms are different. The EC, ELKSS, KBKNR, ECRM, and MNEN methods perform relatively well on this network, with the top 4 nodes identified being consistent with the sequence of the SIR model. PR and PL also achieved acceptable results, with multiple nodes among the top 10 being consistent with the results of the SIR model. However, the BC, CC, and K-shell methods did not perform well, especially the K-shell method. Due to its coarse-grained nature, it could only identify 2 nodes among the top 10 as being consistent with the SIR model.

As shown in Table 4 in the USAir network, the recognition effects of various algorithms are different (**please see lines 435–444 in the revised manuscript**).

According to Table 4, it can be seen that the first two node sequences of KBKNR, BC, CC, GIN, GSM and SIR are consistent. Among them, the recognition effect of KBKNR is relatively ideal, with 8 out of the top 10 nodes being the same as those of SIR. Although the node sequences of MNEN, PL and ECRM methods are not consistent with that of SIR, many of the top 10 nodes they identified are the same as those of SIR, thus they also have certain recognition effects. However, PR and Gravity methods have no consistent nodes with SIR, and the number of nodes that are not the same as the top 10 nodes of SIR is relatively large, so their recognition effects are not very ideal.

As shown in Table 5, in the Powergrid network, the identification effects of various algorithms are different. The first three nodes identified by MNEN are consistent with the SIR model sequence, and among the top 10 nodes, 8 are the same as those in the SIR model. The PL, ECRM, EC, and ELKSS methods do not have any nodes that are consistent with the SIR model sequence, but among the top 10 nodes, more than 6 are the same as those in the SIR model. The identification effects of K-shell, PR, BC, and CC are not very satisfactory, especially for BC and CC. This might be due to the large scale of the network, as none of the top 10 nodes are the same as those in the SIR model (**please see lines 446–461 in the revised manuscript**).

According to Table 7, it can be seen that different infection probabilities have an impact on the Kendall τ ranking consistency of each method.

When the infection probability is 0.01, the KBKNR method performs the best; when the infection probability is within the range of [0.02, 0.05], the MNEN method shows better ranking consistency; when the infection probability is 0.06, both the ELKSS and ECRM methods start to show advantages; when the infection probability is within the range of [0.07, 0.1], the performance of the Gravity, ELKSS, GIN, GSM and ECRM methods is superior to that of the MNEN method (**please see lines 477–484 in the revised manuscript**).

Since KBKNR method cannot be applied to networks with isolated nodes, there is no polyline corresponding to this method in the Protein network in Fig 5, and MNEN method has the best effect; in the other nine networks, MNEN is compared with KBKNR method, and the values of Kendall τ are relatively close.

In Protein, Euroroad, and Powergrid networks, the MNEN method has higher Kendall τ values from 0.01 to 0.1 than other algorithms; in the Dolphins network, it is only lower than ELKSS at 0.1; in the USAir network, the MNEN method is higher than other algorithms from 0.01 to 0.08, lower than ECRM and ELKSS at 0.09, lower than ECRM, ELKSS, and Gravity at 0.1; in the Karate, Email, Ca-GrQc, Ca-Erdos, and Ca-Astroph networks, the recognition effect of the MNEN method is generally average.

The Kendall τ value indicates the consistency between the node sequences obtained by this method and by the SIR model. Therefore, in ten different networks, the performance of MNEN is relatively stable and has achieved a good identification effect (**please see lines 490–502 in the revised manuscript**).

The Karate and Dolphins networks are small, so the infectivity data for each node is displayed directly in Fig 6 In the remaining eight networks, the infectivity data is presented as log10 due to the networks' size. When the curve shows a smooth downward trend from left to right, the higher the consistency between the node importance sequence obtained by each algorithm and the sequence obtained by the SIR model, the less the area of the curve fluctuates. In the Email, Ca-Erdos, Karate, Dolphins, Euroroad, and Powergrid networks, MNEN has the greatest effect; in the Protein network, MNEN is inferior to the ECRM method and is close to that of the ELKSS method; in the Ca-Astroph and USAir networks, the performance of the MNEN method is average compared with the ECRM, ELKSS, and PL methods; in the Ca-GrQc network, the overall curves of all algorithms vary substantially, but the curves of the GSM, GIN, and EC methods have relatively fewer waves, and their performance is slightly better than that of the MNEN method (**please see lines 514–525 in the revised manuscript**).

According to Fig 7 in the Protein, Email, Ca-Erdos, Ca-Astroph, Dolphins, USAir, Euroroad, and Powergrid networks, the MNEN method's first ten nodes have a higher infection rate than other methods. In the Karate network, the BC method has the best overall effect, followed by the MNEN method, while the effects of the other methods are relatively close; in the Ca-GrQc network, MNEN method has a good effect before time step t=2, but the infection effect is lower than BC, CC, and PR beginning at time step t=3, which is close to other methods. Consequently, the MNEN has the greatest overall impact of the ten networks (**please see lines 544–550 in the revised manuscript**).

(2) To enhance the informativeness of the evaluation, we have added the Jaccard similarity coefficient as a new comparison metric. This index quantifies the overlap between the top 15 influential nodes identified by each method and those obtained via the SIR model, offering a complementary perspective. Since this metric does not capture the consistency of node ranking order, we have retained the original Tables 1, 3, 4, and 5. To optimize space and improve readability, we now only list the top 10 nodes in each table. The revised manuscript is as follows:

The Jaccard similarity coefficient is adopted to quantitatively analyze the consistency degree of each algorithm with the SIR model in the identification of key nodes. For a given number of key nodes m, the first m nodes identified by each algorithm form set A, and the first m nodes identified by the SIR model form the reference set B. The calculation formula of the Jaccard similarity coefficient is as follows:

$J\left( A,B \right)=\frac{\left| A\cap B \right|}{\left| A\cup B \right|}$. (11)

The similarity between the recognition results of each algorithm and the SIR model was objectively measured by calculating the ratio of the intersection to the union of the two sets. The Jaccard coefficient ranges from [0, 1], with a value closer to 1 indicating a higher consistency in the key node sets identified by the two methods, and a value closer to 0 indicating a greater difference (**please see lines 388–398 in the revised manuscript**).

First, the influence of nodes in each network is calculated using each method and SIR model, and the node sequence is obtained in descending order. The infection probability α in the SIR model is set to 0.05. Then, the Jaccard similarity coefficient of each algorithm and the SIR model on the top 15 nodes is calculated using formula (11), and finally presented in the form of a heat map, as shown in Fig 4.

Fig 4 illustrates the level of consistency between each algorithm and the SIR model in identifying key nodes across different networks.

**Fig 4. Heatmap of Jaccard similarity between each algorithm and the top 15 nodes of SIR.**

As observed in Fig 4, in the Protein and Powergrid networks, the Jaccard similarity between the top 15 nodes identified by the MNEN method and the SIR model is relatively high, suggesting that the MNEN method effectively identifies key nodes with greater influence within the network, achieving an ideal identification performance. In the Karate network, due to its smaller scale, all algorithms exhibit relatively good identification performance. In the Ca-Erdos, Dolphins, USAir, Euroroad, and Ca-GrQc networks, most algorithms demonstrate satisfactory identification performance. In the Email network, the MNEN method performs slightly less effectively compared to the EC, ELKSS, and ECRM methods. In the Ca-Astroph network, owing to its large scale, the identification performance of all methods is less than optimal. Since this similarity reflects whether the nodes within a certain range are the same, it cannot show whether the node sequences are consistent. Therefore, a table format was adopted to list the node sequences intuitively.

Due to space limitations, here we take the Dolphins, USAir, and Powergrid networks as examples. In Tables 3 - 5, the top 10 nodes obtained by all methods and SIR in these three networks are displayed. For consistency, Tables 3 - 5 all adopt the same color-coding rules as Table 1 (**please see lines 410–433 in the revised manuscript**).

We hope these revisions address your concerns and improve the overall quality and fairness of the manuscript. Thank you again for your valuable feedback.

Yours sincerely,

Feifei Wang, Zejun Sun, Guan Wang, Bohan Sun
